# Countries at risk of importation of chikungunya virus cases from Southern Thailand: A modeling study

**DOI:** 10.1016/j.idm.2019.09.001

**Published:** 2019-09-12

**Authors:** Ashleigh R. Tuite, Alexander G. Watts, Kamran Khan, Isaac I. Bogoch

**Affiliations:** a Dalla Lana School of Public Health, University of Toronto, Toronto, Canada; b BlueDot, Toronto, Canada; c Li Ka Shing Knowledge Institute, St. Michael's Hospital, Toronto, Canada; d Department of Medicine, Division of Infectious Diseases, University of Toronto, Toronto, Canada

**Keywords:** Chikungunya virus, Arboviruses, Travel-related illness, Disease outbreaks, Air travel

## Abstract

Southern Thailand has been experiencing a large chikungunya virus (CHIKV) outbreak since October 2018. Given the magnitude and duration of the outbreak and its location in a popular tourist destination, we sought to determine international case exportation risk and identify countries at greatest risk of receiving travel-associated imported CHIKV cases. We used a probabilistic model to estimate the expected number of exported cases from Southern Thailand between October 2018 and April 2019. The model incorporated data on CHIKV natural history, infection rates in Southern Thailand, average length of stay for tourists, and international outbound air passenger numbers from the outbreak area. For countries highly connected to Southern Thailand by air travel, we ran 1000 simulations to estimate the expected number of imported cases. We also identified destination countries with conditions suitable for autochthonous CHIKV transmission. Over the outbreak period, we estimated that an average of 125 (95% credible interval (CrI): 102–149) cases would be exported from Southern Thailand to international destinations via air travel. China was projected to receive the most cases (43, 95% CrI: 30–56), followed by Singapore (7, 95% CrI: 2–12) and Malaysia (5, 95% CrI: 1–10). Twenty-three countries were projected to receive at least one imported case, and 64% of these countries had one or more regions that could potentially support autochthonous CHIKV transmission. The overall risk of international exportation of CHIKV cases associated with the outbreak is Southern Thailand is high. Our model projections are consistent with recent reports of CHIKV in travelers returning from the region. Countries should be alert to the possibility of CHIKV infection in returning travelers, particularly in regions where autochthonous transmission is possible.

## 1 Introduction

Chikungunya virus (CHIKV) is an arbovirus transmitted to humans by the bite of infected *Aedes* mosquitoes (Evans and Kaslow, 1997). Symptoms of infection include fever and severe join pain (Evans and Kaslow, 1997). The first reported cases of CHIKV infection in Thailand were in 1958 (Hammon, Rudnick, & Sather, 1960), and sporadic cases and outbreaks have been reported throughout the country since that time (Rianthavorn et al., 2010; Thaikruea et al., 1997). Prior to 2008, circulating strains were of the Asian lineage (Thaikruea et al., 1997). In 2008–2010, there was a large outbreak that was first reported in Narathiwat province in the south of the country, near the Malaysian border (Rianthavorn et al., 2010). The outbreak eventually spread to more than one-third of districts across Thailand and was associated with the novel introduction of the East Central and South African lineage to the country (Chadsuthi et al., 2018). A subsequent serosurvey conducted in 2014 demonstrated the wide extent of CHIKV transmission in the southern provinces of Trang and Narathiwat, where age-standardized seroprevalence was estimated at 29.6% (Vongpunsawad, Intharasongkroh, Thongmee, & Poovorawan, 2017).

The first large CHIKV outbreak in Thailand since 2008–2010 has been occurring since October 2018. Cases are concentrated in Southern Thailand, a region of the country that includes popular tourist destinations, such as Phuket and Krabi. Given the size of the outbreak and its co-localization to popular vacation destinations, there is concern that there will be cases exported to new destinations, some of which may have environmental conditions that are suitable for autochthonous CHIKV transmission. Indeed, imported cases in tourists returning from Thailand have already been reported (Javelle et al., 2019; Kantele, 2019) and one of the countries with an imported case (France) has previously reported autochthonous transmission of CHIKV following a travel-related infection (Grandadam et al., 2011).

Given these concerns, we used a model-based approach to estimate the overall risk of exported CHIKV cases globally and to identify countries at greatest risk of receiving cases associated with the outbreak in Thailand.

## 2 Methods

### 2.1 Case data for Southern Thailand

**Fig. 1:**
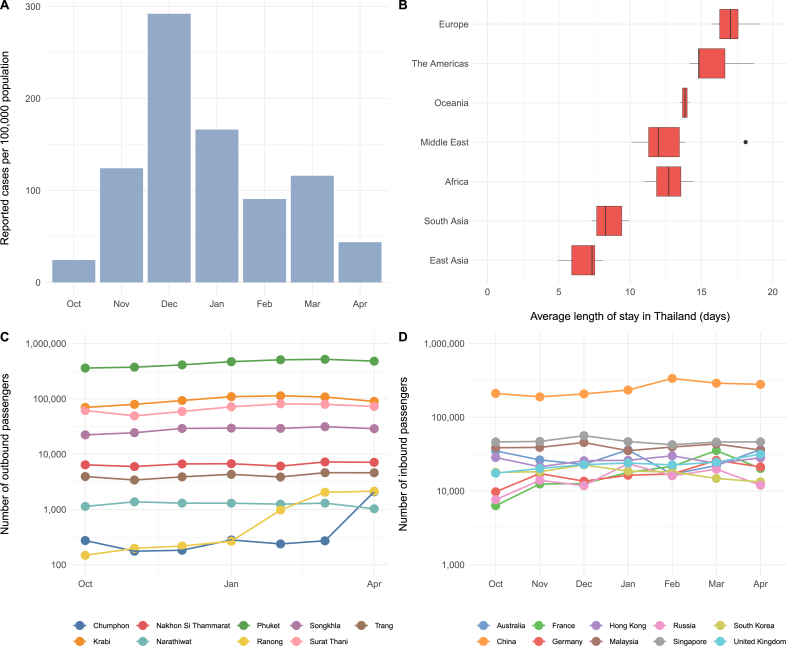
**Overview of key model inputs.** (A) Annualized monthly reported chikungunya virus cases per 100,000 population in Southern Thailand, October 2018 to April 2019. (B) Average length of stay by international visitors to Thailand in 2017, by origin region, as reported by Thailand's Ministry of Tourism and Sports (Department of Tourism, ). (C) Monthly outbound passengers from Southern Thailand, by province. (D) Monthly inbound passengers from Southern Thailand, for the top 10 destination countries. Air passenger numbers are for October 2017 to April 2018 and are plotted on a logarithmic scale.

We obtained publicly-available cumulative reported case rates from Thailand's Bureau of Epidemiology (Bureau of Epidemiology, 2019a). Reports are generally published weekly, with rates reported by administrative region (North, Northeastern, Central, and South). To date, rates are highest in the South, which includes 14 provinces and covers an area of approximately 70,715 km^2^. We restricted the analysis to this region. Rates were converted to cumulative cases using 2017 population estimates (Thailand Ministry of Public Health, 2008), and were then converted to monthly rates (Fig. 1).

### 2.2 International outbound air passengers from Southern Thailand

We identified all airports in the 14 provinces in the Southern Thailand administrative region and obtained the total number of outbound passengers from these airports to all countries from the International Air Transport Association (International Air Transport Association, 2017), for the years 2017–2018 (the most recent years available) (Fig. 1). We included all passengers traveling via direct and indirect flights to each final destination country. We focused on the months October to April, to align with the outbreak data. We selected the top 25 countries based on outbound travel volumes from the region over this time period.

### 2.3 Exportation risk model

We used a previously described modeling approach (Tuite et al., 2019; Lewnard, Gonsalves, & Ko, 2016) to quantify the risk of travel-associated CHIKV case exportation from Thailand. This method estimates the probability that an individual traveling from the outbreak region will acquire infection and export it to another country. Key model inputs are displayed graphically in Fig. 1 and parameters are described in detail Table 1. We incorporated data on CHIKV natural history, infection rates in Southern Thailand, and international outbound passenger volumes from the outbreak area, to quantify the expected number of exported cases over the October to April outbreak period. To account for the fact that the air travel data were not contemporaneous with the outbreak data, we assumed the monthly passengers to each country were normally distributed with mean equal to the observed value and a standard deviation of 10% of the mean. Residents and visitors to Thailand were treated separately, to account for different infection exposure windows, based on total time spent in the region prior to international travel. We used data from the World Tourism Organization to estimate the proportion of passengers from Thailand that are visitors (World Tourism Organization, 2019). We also obtained average length of time spent in Thailand by international visitors by country of origin for the year 2017, using regional averages for length of stay when country-specific estimates were unavailable (due to low numbers of travelers to Thailand) (Fig. 1). (Department of Tourism, ) Residents were assumed to travel at the mid-point of the month and also had the possibility of being exposed to infection at any point in the prior month. Individuals could be infected at any point during their length of stay in the country. Latent (mean 5 days, standard deviation (s.d.) 1.2 days) and infectious (mean 4 days, s.d. 0.6 days) periods were assumed to follow lognormal distributions (Centers for DIsease Control and Prevention, 2018). We assumed that 90% of CHIKV infections were symptomatic (Centers for DIsease Control and Prevention, 2018).

**Table 1:** Model input parameters.

Parameter	Value	Details	Source
Annualized monthly incidence in Southern Thailand (per 100,000 population)	Oct: 24.3 Nov: 124.1 Dec: 292.1 Jan: 166.2 Feb: 90.7 Mar: 115.9 Apr: 43.7	Monthly rates of reported cases are assumed to represent CHIKV incidence	Bureau of Epidemiology (2019b); Thailand Ministry of Public Health (2008)
Probability case is symptomatic	0.9		Centers for Disease Control and Prevention (2018)
Monthly number of outbound passengers departing Southern Thailand (thousands)	Oct: 526.1 Nov: 538.1 Dec: 605.0 Jan: 693.9 Feb: 744.3 Mar: 753.2 Apr: 688.7	Normal distribution assumed; mean is monthly outbound passengers from South Thailand to all international destinations; assume the s.d. Is 10% of the monthly passenger volume; country-specific numbers were used for country-level risk estimates	International Air Transport Association (2017)
Exposure duration, tourist (days)	Variable, range: 5-19	Average time spent in Thailand by tourists; dependent on tourist origin country	Department of Tourism
Exposure duration for month of travel, resident (days)	15	Thai residents are assumed to travel at the midpoint of the month. The exposure window for residents is assumed to extend back an additional 30 days to the prior month.	Assumption
Proportion of outbound passengers that are residents of Thailand	0.2		World Tourism Organization (2019)
Latent period (days)	5 (s.d. 1.2)	Log-normal distribution	Centers for Disease Control and Prevention (2018)
Infectious period (days)	4 (s.d. 0.6)	Log-normal distribution	Centers for Disease Control and Prevention (2018)

Abbreviations: CHIKV – chikungunya virus; s.d. – standard deviation.

We calculated the probability of infection for a given month as:

$$1-\;e^{-\left(\frac{symptomatic\;incidence}{probability\;symptomatic}\;\;\times \;\frac{exposure\;duration}{365}\right)}$$

The expected number of infected travelers to each destination country was calculated assuming a binomial distribution: binomial (n = number of monthly passengers, p = monthly probability of infection). For travelers to a given destination country, each infected individual was assigned a latent (time from infection to onset of infectiousness) and infectious (time during which a person is at risk of transmitting the virus) period, drawn from distributions (Table 1), and time of infection (drawn from uniform distribution: unif (0, exposure duration)). If the summed time of infection, latent, and infectious durations were greater than the time spent in the country, this represented an exported case.

For each of the 25 countries with the greatest travel volumes from Southern Thailand, we ran 1000 simulations for each monthly time point. We summed the expected number of imported cases over the outbreak time period and report the mean and 95% credible intervals for each country.

### 2.4 Identification of countries with suitability for autochthonous CHIKV transmission

We used a previously published CHIKV suitability map to identify countries with regions where autochthonous CHIKV transmission was considered possible, based on suitable environmental conditions and the presence of competent mosquitoes (Nsoesie et al., 2016). A country was considered suitable if it contained one or more 5 × 5 km regions where the probability of conditions suitable to support mosquito-borne CHIKV transmission was greater than 0.5.

## 3 Results

The chikungunya outbreak in Thailand is ongoing, with a total of 3319 cases reported across the country between 1 January and 5 May 2019 (Bureau of Epidemiology, 2019). By comparison, 23 cases were reported over the same period in 2018 (Bureau of Epidemiology, 2018). To date, reported rates of CHIKV infection during the current outbreak were highest in December 2018.

Between October 2017 and April 2018, there was an average of 649,907 monthly outbound passengers departing from airports in Southern Thailand, with the greatest number of travelers departing in March. Within the region, Phuket was the most popular departure location (Fig. 1). China was the most popular international destination for outbound flights from Southern Thailand, accounting for 38% of all international passengers over this time period.

Over the outbreak period, we estimated that an average of 125 (95% CrI: 102–149) cases would be exported from Southern Thailand to international destinations via air travel. China was projected to receive the most cases (43, 95% CrI: 30–56), followed by Singapore (7, 95% CrI: 2–12) and Malaysia (5, 95% CrI: 1–10) (Table 2). 64% of countries projected to receive a mean of one or more imported cases had one or more regions identified as potentially suitable for autochthonous transmission.

**Table 2:** Estimated number of imported chikungunya virus infected travelers, based on reported cases in Southern Thailand, October 2018 to April 2019.

Destination	Expected number of imported cases (95% CrI)	Travel volume rank	Ongoing transmission possible?^a^
China	42.7 (30–56)	1	Yes
Singapore	6.7 (2–12)	2	Yes
Malaysia	5.4 (1–10)	3	Yes
Australia	5.1 (1–10)	4	Yes
United Kingdom	4.5 (1–9)	6	No
Hong Kong	3.9 (1–8)	5	Yes
France	3.5 (1–8)	7	Yes
South Korea	3.3 (0–7)	8	No
Germany	3.2 (0–7)	9	No
Russia	3.1 (0–7)	10	No
Sweden	2.6 (0–6)	12	No
India	2.3 (0–5)	11	Yes
Cambodia	1.8 (0–5)	13	Yes
Denmark	1.8 (0–5)	19	No
Italy	1.8 (0–5)	18	Yes
Japan	1.8 (0–5)	15	Yes
Finland	1.7 (0–4)	21	No
Switzerland	1.7 (0–5)	17	No
Indonesia	1.4 (0–4)	16	Yes
United States	1.4 (0–4)	22	Yes
Viet Nam	1.4 (0–4)	14	Yes
Myanmar	1.3 (0–4)	20	Yes
Macao	1 (0–3)	23	Yes
Belgium	0.7 (0–3)	25	No
United Arab Emirates	0.7 (0–3)	24	Yes
Total	124.6 (102–149)	–	–

a Based on one or more regions in the country that could support autochthonous transmission with probability greater than 0.5.

The number of expected imported cases generally aligned with travel volumes, with countries receiving more travelers expected to receive more cases (Table 2). However, the inclusion of average length of stay for tourists to Southern Thailand did influence the results. For instance, Hong Kong is ranked fifth for outbound passengers from the region but was expected to receive fewer exported cases than lower ranked United Kingdom, due to the shorter length of stay for visitors from Hong Kong compared to the United Kingdom.

## 4 Discussion

We used a model-based approach to quantify the risk of international exportation of CHIKV cases associated with the current outbreak in Southern Thailand. Between October 2018 and April 2019, the overall exportation risk was high, with close to 125 exported cases projected. Many countries were at risk for receiving one or more travel-associated cases, with more than 30% of imported cases expected to occur in travelers to China. Notably, the availability and incorporation of length of stay data allowed for more refined estimates of importation risk than would be obtained using travel volumes alone.

Recent reports of cases of CHIKV in travelers returning from Southern Thailand are consistent with our model projections (Javelle et al., 2019; Kantele, 2019). Of the seven countries reporting imported cases in these reports, five (United Kingdom, France, Sweden, Switzerland, and Finland) were identified in our model as likely to import at least one case. Romania and Israel are countries that were not identified by our model as being among the highest risk countries, despite both countries reporting imported cases (Javelle et al., 2019). The model estimated numbers of imported cases were low, but not zero, at 0.2 (95% CrI: 0–1) and 0.4 (95% CrI: 0–2) cases for Israel and Romania, respectively. Although returning travelers can serve as sentinels of disease activity (Lau, Weinstein, & Slaney, 2013; Wilder-Smith & Boggild, 2018), the absence of cases in returning travelers in locations where risk of importation is considered high may serve to highlight locations where surveillance and/or notification systems are less robust.

While the current data suggest that the outbreak peaked in December and may be waning, it is worth noting that the previous CHIVK outbreak in Thailand had two distinct temporal peaks in January and June of 2009, with the timing of the peaks corresponding with peaks in rainfall (Chadsuthi, Iamsirithaworn, Triampo, & Cummings, 2016). Climatic variation has been shown to influence transmission (Chadsuthi et al., 2016) and we may see increased cases with the return of the rainy season in mid-May. As such, countries with large number of travelers to Southern Thailand should remain alert to the possibility of CHIKV infection in returning travelers, particularly in regions where autochthonous transmission is possible.

Our approach for estimating importation risk has several limitations. The air travel data are not contemporaneous with the outbreak data and therefore may not have captured possible changes in air travel patterns from Southern Thailand. For this analysis, we focused our analysis on the south region of the country, since this is where the majority of reported cases are occurring. The publicly-available data used in the analysis did not allow for investigation of expected case exportation from the individual Thai provinces. Additionally, reliance on reported cases likely underestimates the true burden of infection, since not all infected individuals will present to medical care or have their case reported. By restricting the analysis to passengers with international travel itineraries that departed from airports in the southern region of Thailand (including both direct and indirect flights), we excluded passengers who may have visited Southern Thailand over the course of their travels but departed from airports located in other parts of the country. We reasoned that including all outbound passengers from across the country would overestimate the at-risk population but acknowledge that the model-derived number of travelers at risk is likely a conservative estimate. Finally, our model does not account for lags in seeking medical care or case reporting. Although most patients will recover from their infection within a week, symptoms may persist for months, such that cases could be diagnosed in their destination country when they are no longer viremic, and thus no longer pose a risk for sustained transmission.

In summary, we have demonstrated that the 2018–2019 chikungunya outbreak in Southern Thailand has been associated with a high risk of international case exportation. Our model projections are consistent with recent reports of CHIKV in travelers returning from the region. Countries should be alert to the possibility of CHIKV infection in returning travelers, particularly in regions where autochthonous transmission is possible.

## Author contributions

ART and AGW contributed to the design and analysis of the study, as well as the preparation of the manuscript. KK and IIB made significant content contributions and edits to the manuscript.

## Funding

IIB is supported by the Tesari Charitable Foundation and the Ricker Family Foundation.

## Conflict of interest/disclosure

KK is the founder of BlueDot, a social enterprise that develops digital technologies for public health. All authors received employment or consulting income from BlueDot during this research.
